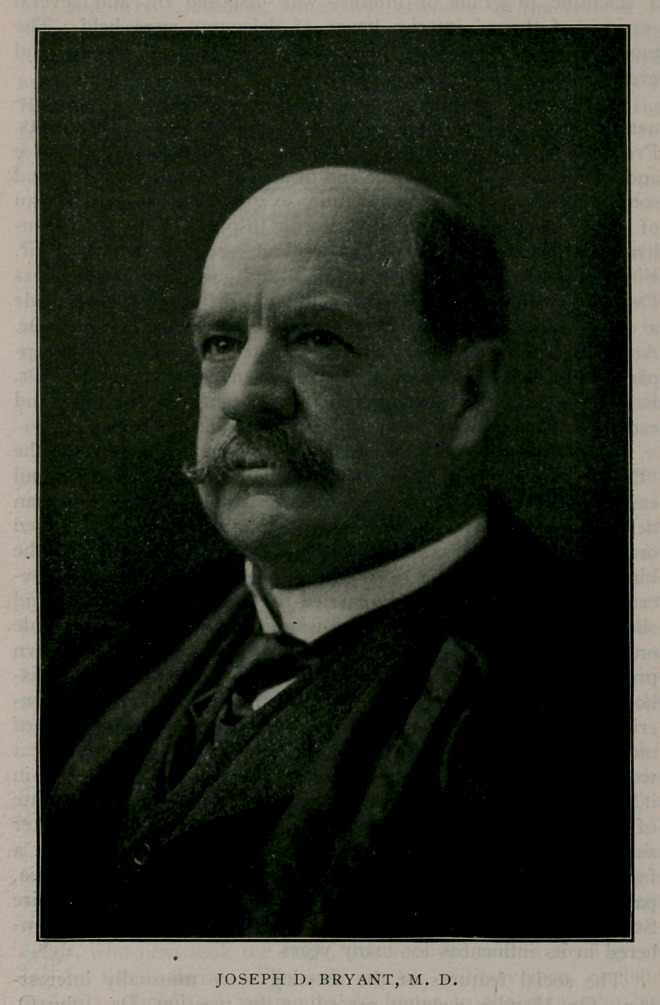# The Centennial of a Medical Society

**Published:** 1906-02

**Authors:** 


### The Centennial of a Medical Society.

WE have made occasional reference in these columns during the past two or three years to the fact that the Medical Society of the State of New York, at its annual meeting in 1906, woud celebrate the completion of the first century of its existence. This memorable occasion was adequately observed at Albany, January 30, 31, and February 1, 1906, under the administration of Dr. Joseph D. Bryant of New York, supported by an able cabinet of subordinate officers.

It is appropriate that some of the special features of this interesting event should be enumerated. The president's address dealt most happily with the history and purpose of the society, and the results accomplished; with the division in its ranks of 1882-4; and the final reunion of 1906. Perhaps no member has worked more zealously or to greater purpose in the interests of medical unity than President Bryant, and his continuance in office to complete the plan of unification is a fitting recognition of his inestimable services.

A marked feature of the occasion was a public address by ex-President Grover Cleveland, delivered on the evening of the first day, during which he referred to his pleasant visit to the scenes of his former labors as Governor, and then the medical profession was discoursed to and of in a pleasant fashion. Other addresses during the progress of the meeting were made by Dr. L. S. McMurtry, president of the American Medical Association; Dr.

William W. Keen, of Philadelphia; and Dr. William H. Welch, of Baltimore.

JOSEPH D. BRYANT, M. D.

Orations were presented by Dr. Samuel B. Ward, on Medicine; Dr. Herman M. Briggs, on Sanitation; and Dr. Roswell Park, on Surgery. An abstract of the latter prepared by the author is published in the present issue of the Journal. Besides these, a scientific program of interest was disposed of, and several sessions of the ad interim house of delegates were held. The mornings were devoted to the scientific work; the afternoons and evenings to the other exercises.

The evening of Wednesday was set apart for the annual dinner, which was served in Odd Fellow's Hall to about 450 guests. President Bryant took the Chair at 7.30 o'clock, and when coffee and cigars were reached at the end of an elaborate ménu, he found some difficulty in securing quietude, so great was the enthusiasm of the assemblage. The music was inspiring, the songs enlivening, and the gathering was jubilant,—reunion filled the air. Finally, after the exercise of patience and good natured firmness Dr. Bryant obtained control of his coltish banqueters, and made a clever little speech, at the close of which he introduced Dr. Arthur G. Root, of Albany, as toastmaster. It soon became apparent that the mantle of office had fallen upon a master. Dr. Root presented the speakers in a most felicitous manner, and each felt the inpiration of the introduction.

Lieutenant-Governor M. Linn Bruce, in responding to the "Empire State," made a masterful speech, full of thought, and excellent teaching. It was one of the best we ever heard a layman deliver to a company of doctors. He is an exceptionally gifted orator and when he said "The government you have is just the kind of government you deserve, because you can have any government you want," it fairly carried the audience to its feet, and shouts of applause followed. Bishop Doane, of Albany, made one of his most delightful speeches in responding for his own profession, and it was a genuine pleasure to listen to the good bishop in his happiest ein. Dr. L. S. McMurtry, president of the American Medical Associatiovn, spoke of that great organised body of medical men in his usual felicitious manner, describing its great work, mentioning the adantages belonging to membership in it, and eloquently congratulating the Medical Society of the State of New York upon its return to representation in its daughter association. Dr. William H. Welch, of Baltimore, also made a forceful and able speech and Dr. W. W. Keen, of Philadelphia, paid eloquent tribute to the reunited profssion of the Empire State. All in all, it was a great occasion and will be remembered in its influences for many years.

The social features of the meeting were unusually interesting. On Monday evening preceding the meeting, Dr. John O. Roe and Dr. F. C. Curtis entertained the president and ex-presidents of the society, and the joint committee of conference, at dinner at the Fort Orange Club. Dr. and Mrs. Samuel B. Ward, on the same evening gave a brilliant reception in honor of Mr. and Mrs. Cleveland, which was attended by the fashion of Albany as well as the visitors to the society. Other interesting functions comprised a dinner by Dr. and Mrs. James P. Boyd and a luncheon at the Albany Club by Dr. Willis G. Macdonald. It is evident from this very meagre and cursory account that the centenary of the society was properly observed and that reunion is established on a solid and comprehensive basis.

## Figures and Tables

**Figure f1:**